# ﻿A survey of megachilid bees of the Nakhchivan Autonomous Republic of Azerbaijan, with description of a new species of *Pseudoanthidium* Friese, 1898 (Hymenoptera, Megachilidae)

**DOI:** 10.3897/zookeys.1262.173271

**Published:** 2025-12-05

**Authors:** Alexander V. Fateryga, Mahir M. Maharramov, Maxim Yu. Proshchalykin

**Affiliations:** 1 T.I. Vyazemsky Karadag Scientific Station – Nature Reserve of RAS – Branch of A.O. Kovalevsky Institute of Biology of the Southern Seas of RAS, Nauki Str. 24, Kurortnoye, 298188 Feodosiya, Russia Vyazemsky Karadag Scientific Station Feodosiya Russia; 2 Nakhchivan State University, University Campus, AZ 7012 Nakhchivan, Azerbaijan Nakhchivan State University Nakhchivan Azerbaijan; 3 Federal Scientific Center of the East Asia Terrestrial Biodiversity, Far Eastern Branch of the Russian Academy of Sciences, 100-let Vladivostoku Ave. 159, 690022 Vladivostok, Russia Federal Scientific Center of the East Asia Terrestrial Biodiversity, Far Eastern Branch of the Russian Academy of Sciences Vladivostok Russia

**Keywords:** Biodiversity, Caucasus, new record, Palaearctic region, taxonomy

## Abstract

A list of 184 species of megachilid bees from 19 genera and five tribes is reported for the Nakhchivan Autonomous Republic of Azerbaijan. The list is based on 2,099 examined specimens identified to 171 species; 13 additional species are known on the base of literature records only. A new species, Pseudoanthidium (Exanthidium) astafurovae Fateryga, Maharramov & Proshchalykin, **sp. nov.**, is described. Anthidium (Anthidium) akanthurum Nadimi & Talebi, 2014, **sp. resurr.**, is recognised as a distinct species, not a synonym of A. (A.) gussakovskiji Mavromoustakis, 1939. Nineteen species are new to Azerbaijan: A. (A.) akanthurum, Chelostoma (Foveosmia) foveolatum (Morawitz, 1868), Coelioxys (Allocoelioxys) argenteus Lepeletier de Saint-Fargeau, 1841, C. (A.) obtusus Pérez, 1884, C. (Coelioxys) quadridentatus (Linnaeus, 1758), Heriades (Michenerella) punctulifera Schletterer, 1889, Hoplitis (Alcidamea) brachypogon (Pérez, 1879), H. (A.) campanularis (Morawitz, 1877), H. (Anthocopa) mocsaryi (Friese, 1895), Megachile (Eutricharaea) anatolica Rebmann, 1968, M. (Megachile) genalis Morawitz, 1880, *Metadioxys graeca* (Mavromoustakis, 1963), Osmia (Helicosmia) breviata Warncke, 1988, O. (H.) clypearis acuta Warncke, 1988, O. (Osmia) scheherazade Peters, 1978, O. (Tergosmia) rhodoensis (van der Zanden, 1983), Protosmia (Nanosmia) magnicapitis (Stanek, 1969), Pseudoanthidium (Pseudoanthidium) rhombiferum (Friese, 1917), and P. (P.) syriacum Kasparek, 2024. *Metadioxys formosus* (Morawitz, 1875) and Coelioxys (Allocoelioxys) sogdianus Morawitz, 1875 are excluded from the fauna of Azerbaijan. The number of species of Megachilidae known from Azerbaijan is increased to 196.

## ﻿Introduction

**Figure 1.**
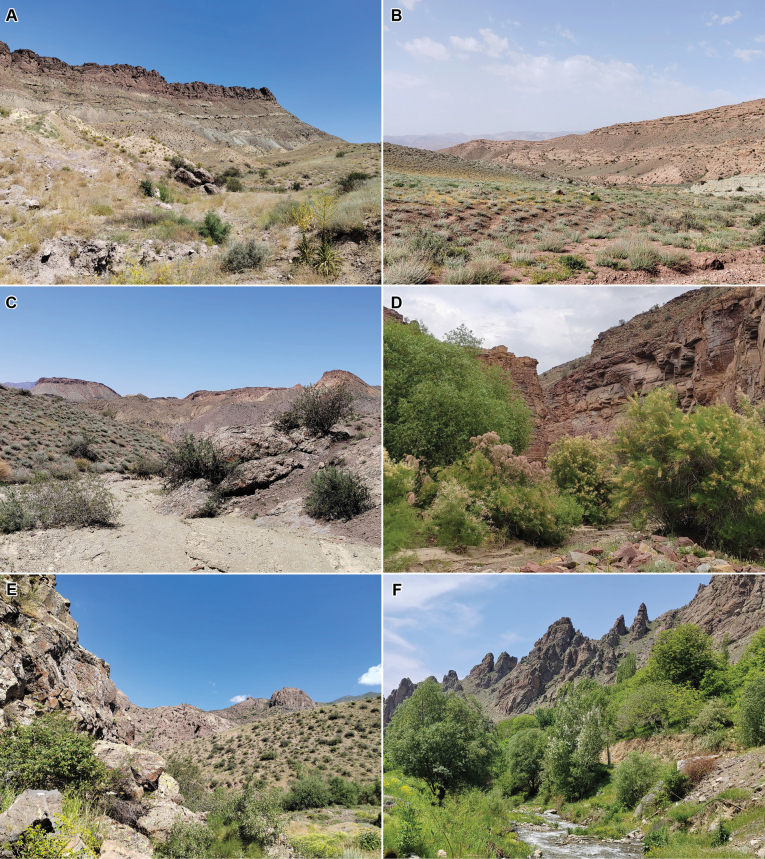
Landscapes of the Nakhchivan Autonomous Republic of Azerbaijan. A. Dry slopes with sparse herbaceous vegetation and flowering *Verbascum pyramidatum* M. Bieb. (on the right) at Julfa, Daridagh-2, 1100 m, 20.VI.2024; B. Wormwood (*Artemisia* sp.) mountain semi-desert at Julfa, Daridagh-2, 1100 m, 19.V.2025; C. Community of *Rhamnus erythroxyloides* Hoffmanns. at Ordubad, Bilav, 1050 m, 22.V.2025; D. Community of *Tamarix* sp. at Ordubad, Bilav, 1050 m, 27.V.2025; E. Community of various shrubs at Shakhbuz, Badamli, 1290 m, 21.VI.2024; F. River valley at Ordubad, Unus, 1680 m, 24.V.2025.

The Megachilidae is the third largest bee family numbering more than 4,100 described species worldwide (Michener 2007; Ascher and Pickering 2025). The Nakhchivan Autonomous Republic (AR) is a landlocked exclave of Azerbaijan, with the area of about 5,500 km^2^. The territory of the autonomy is located within the Lesser Caucasus at altitudes of about 600–3,905 m a.s.l. (Maharramov 2015). Landscapes of the Nakhchivan AR are mostly xeric herb and shrub communities, with just a few trees along river valleys (Fig. 1), which are known as favourable for the bee diversity (Orr et al. 2020). In recent years, the megachilid bee fauna of the autonomy received a special attention. Several faunistic papers were published (Proshchalykin et al. 2019; Fateryga et al. 2020; Proshchalykin and Maharramov 2020; Maharramov et al. 2021, 2023), increasing the number of species known from the Nakhchivan AR to 160. At the same time, the number of species known from the whole of Azerbaijan was increased to 174, which was expected to be still very far from the true number of species occurring in the republic. However, new material collected in the autonomy in 2024 and 2025 contained numerous additional species new to Azerbaijan, as well as an undescribed species. The purpose of the present contribution is to publish a complete list of all species of megachilid bees known from the Nakhchivan AR to date.

## ﻿Material and methods

Several field expeditions were made to various districts of the Nakhchivan Autonomous Republic of Azerbaijan in 2018 and 2019 by M. Proshchalykin, Kh. Aliyev, and M. Maharramov, in 2020–2022 by M. Maharramov, and in 2024 and 2025 by M. Proshchalykin and M. Maharramov. Collected specimens are deposited mainly in the collections of the Federal Scientific Center of the East Asia Terrestrial Biodiversity of the Far Eastern Branch of the Russian Academy of Sciences, Vladivostok, Russia (**FSCV**, registration number 2797657) and the research collection of A.V. Fateryga, Feodosiya, Russia (**CAFK**). Old material deposited in the Institute of Zoology of the National Academy of Sciences of Azerbaijan, Baku, Azerbaijan (**IZAB**) and the Zoological Institute of the Russian Academy of Sciences, Saint Petersburg, Russia (**ZISP**) was also studied. A total of 2,209 specimens of megachilid bees from the Nakhchivan AR were examined. Selected specimens were sent to be deposited (some of them temporary) in the Entomological Collection of ETH Zurich, Switzerland (**ETHZ**), Muséum d’Histoire Naturelle de Neuchâtel, Switzerland (**MHNN**), and the research collections of M. Kasparek, Heidelberg, Germany (**CMKH**). Possible literature sources were also studied. Specimens were identified mostly using a reference collection (CAFK), as well as following keys by Kasparek (2022b, 2024), Bogusch (2023), Praz and Bénon (2023), and Müller (2025a). Many species of the tribe Osmiini were identified by A. Müller (Zurich, Switzerland). General distribution of species reported as new to Azerbaijan is based on Müller (2025b) for the tribe Osmiini, as well as Proshchalykin and Fateryga (2017), Bogusch (2023), Proshchalykin et al. (2023), Kasparek (2024), and Ascher and Pickering (2025) for other taxa.

## ﻿Results

As the result of the study, 2,099 specimens of megachilid bees from the Nakhchivan Autonomous Republic of Azerbaijan were identified to 171 species. The remaining 110 specimens belonging to the tribes Osmiini and Megachilini unfortunately remained unidentified. Thirteen more species were added to the list of the megachilid bees known from the Nakhchivan AR on the base of literature records only, their occurrence in the autonomy require further confirmation. Thus, a total of 184 species from 19 genera and five tribes were revealed occurring in the Nakhchivan AR. Full label data of all specimens are represented in Suppl. material 1. One species is new to science, while 19 species are new to Azerbaijan; their full label data and general distribution are listed below. Besides them, three other species are new to the Nakhchivan AR: Pseudoanthidium (Pseudoanthidium) stigmaticorne (Dours, 1873), Stelis (Stelidomorpha) nasuta (Latreille, 1809), and S. (Stelis) phaeoptera (Kirby, 1802) (Table 1).

**Table 1.** A list of the megachilid bees of the Nakhchivan Autonomous Republic of Azerbaijan (species new to the autonomy are indicated with an asterisk; species new to Azerbaijan are indicated with two asterisks).

Species name	Literature records	Material examined
**Tribe Lithurgini**		
*Lithurgus chrysurus* Fonscolombe, 1834	Maharramov et al. (2014, 2021)	124 ♀, 75 ♂
*Lithurgus cornutus* (Fabricius, 1787)	Maharramov et al. (2014, 2021)	36 ♀, 34 ♂
*Lithurgus tibialis* Morawitz, 1875	Maharramov et al. (2023)	13 ♂
**Tribe Osmiini**		
Chelostoma (Chelostoma) emarginatum (Nylander, 1856)	Proshchalykin and Maharramov (2020)	2 ♀, 1 ♂
Chelostoma (Chelostoma) mocsaryi Schletterer, 1889	Maharramov et al. (2014)	–
Chelostoma (Foveosmia) distinctum (Stöckhert, 1929)	Proshchalykin and Maharramov (2020)	6 ♀, 41 ♂
Chelostoma (Foveosmia) foveolatum (Morawitz, 1868)**	–	4 ♀, 2 ♂
Chelostoma (Foveosmia) garrulum (Warncke, 1991)	Proshchalykin et al. (2019); Proshchalykin and Maharramov (2020); Ascher and Pickering (2025)	11 ♀, 51 ♂
Chelostoma (Gyrodromella) orientale Schletterer, 1890	Proshchalykin and Maharramov (2020)	16 ♀, 17 ♂
Chelostoma (Gyrodromella) rapunculi (Lepeletier de Saint-Fargeau, 1841)	Maharramov et al. (2014), as *Ch. proximum*; Proshchalykin and Maharramov (2020)	7 ♀, 8 ♂
Heriades (Heriades) crenulata Nylander, 1856	Maharramov et al. (2014)	–
Heriades (Heriades) rubicola Pérez, 1890	Maharramov et al. (2023)	5 ♀, 6 ♂
Heriades (Heriades) truncorum (Linnaeus, 1758)	Maharramov et al. (2014, 2023)	6 ♀, 1 ♂
Heriades (Michenerella) punctulifera Schletterer, 1889**	–	2 ♀
Hoplitis (Alcidamea) acuticornis (Dufour & Perris, 1840)	Proshchalykin and Maharramov (2020)	3 ♀, 1 ♂
Hoplitis (Alcidamea) brachypogon (Pérez, 1879)**	–	1 ♂
Hoplitis (Alcidamea) campanularis (Morawitz, 1877)**	–	2 ♀
Hoplitis (Alcidamea) eburnea (Warncke, 1991)	Proshchalykin and Maharramov (2020)	1 ♀, 2 ♂
Hoplitis (Alcidamea) fulva (Eversmann, 1852)	Proshchalykin and Maharramov (2020)	19 ♀, 6 ♂
Hoplitis (Alcidamea) leucomelana (Kirby, 1802)	Maharramov et al. (2014); Proshchalykin and Maharramov (2020)	6 ♀, 5 ♂
Hoplitis (Alcidamea) mollis Tkalců, 2000	Proshchalykin et al. (2019); Ascher and Pickering (2025)	3 ♀
Hoplitis (Alcidamea) tridentata (Dufour & Perris, 1840)	Proshchalykin et al. (2019); Ascher and Pickering (2025)	6 ♀, 1 ♂
Hoplitis (Anthocopa) bisulca (Gerstäcker, 1869)	Proshchalykin and Maharramov (2020)	4 ♀, 6 ♂
Hoplitis (Anthocopa) dalmatica (Morawitz, 1871)	Proshchalykin et al. (2019); Ascher and Pickering (2025)	1 ♀
Hoplitis (Anthocopa) fasciculata (Alfken, 1934)	Proshchalykin et al. (2019); Ascher and Pickering (2025)	3 ♀, 6 ♂
Hoplitis (Anthocopa) grumi (Morawitz, 1894)	Proshchalykin and Maharramov (2020)	4 ♀, 1 ♂
Hoplitis (Anthocopa) jakovlevi (Radoszkowski, 1874)	Maharramov et al. (2014)	–
Hoplitis (Anthocopa) mocsaryi (Friese, 1895)**	–	1 ♀
Hoplitis (Anthocopa) perezi (Ferton, 1895)	Proshchalykin et al. (2019); Proshchalykin and Maharramov (2020); Ascher and Pickering (2025)	5 ♀
Hoplitis (Anthocopa) yermasoyiae asiae (Tkalců, 1979)	Proshchalykin and Maharramov (2020)	1 ♀
Hoplitis (Hoplitis) adunca (Panzer, 1798)	Maharramov et al. (2014)	2 ♀
Hoplitis (Hoplitis) annulata crenulata (Morawitz, 1871)	Proshchalykin and Maharramov (2020)	27 ♀, 28 ♂
Hoplitis (Hoplitis) astragali Fateryga, Müller & Proshchalykin, 2023	Fateryga et al. (2023); Ascher and Pickering (2025)	4 ♀
Hoplitis (Hoplitis) flabellifera (Morice, 1901)	Proshchalykin and Maharramov (2020)	3 ♀
Hoplitis (Hoplitis) manicata (Morice, 1901)	Proshchalykin and Maharramov (2020)	19 ♀, 26 ♂
Hoplitis (Hoplitis) mutica (Warncke, 1991)	Proshchalykin and Maharramov (2020)	2 ♀, 1 ♂
Hoplitis (Hoplitis) parasitica (Warncke, 1991)	Maharramov et al. (2023)	2 ♀
Hoplitis (Hoplitis) strymonia Tkalců, 1999	Maharramov et al. (2023)	5 ♀
Hoplitis (Pentadentosmia) laevifrons (Morawitz, 1872)	Maharramov et al. (2014)	–
Hoplitis (Pentadentosmia) rufopicta (Morawitz, 1875)	Maharramov et al. (2023)	1 ♂
Hoplitis (Pentadentosmia) tringa (Warncke, 1991)	Proshchalykin and Maharramov (2020)	2 ♀, 2 ♂
Osmia (Allosmia) melanura Morawitz, 1871	Proshchalykin and Maharramov (2020); Ascher and Pickering (2025)	8 ♀
Osmia (Allosmia) rufohirta Latreille, 1811	Proshchalykin and Maharramov (2020); Ascher and Pickering (2025)	2 ♀
Osmia (Erythrosmia) andrenoides Spinola, 1808	Proshchalykin et al. (2019); Proshchalykin and Maharramov (2020); Ascher and Pickering (2025)	13 ♀, 15 ♂
Osmia (Helicosmia) aeruginosa Warncke, 1988	Proshchalykin and Maharramov (2020)	1 ♀
Osmia (Helicosmia) aurulenta Panzer, 1799	Maharramov et al. (2014)	12 ♀
Osmia (Helicosmia) breviata Warncke, 1988**	–	1 ♀
Osmia (Helicosmia) caerulescens (Linnaeus, 1758)	Maharramov et al. (2014); Proshchalykin and Maharramov (2020)	19 ♀, 10 ♂
Osmia (Helicosmia) cinerea Warncke, 1988	Proshchalykin and Maharramov (2020)	3 ♀
Osmia (Helicosmia) clypearis acuta Warncke, 1988**	–	4 ♀
Osmia (Helicosmia) dimidiata Morawitz, 1870	Proshchalykin et al. (2019); Proshchalykin and Maharramov (2020); Ascher and Pickering (2025)	19 ♀, 6 ♂
Osmia (Helicosmia) dives Mocsary, 1877	Proshchalykin et al. (2019); Ascher and Pickering (2025)	12 ♀, 3 ♂
Osmia (Helicosmia) labialis Pérez, 1879	Proshchalykin et al. (2019)	2 ♀
Osmia (Helicosmia) leaiana (Kirby, 1802)	Maharramov et al. (2014, 2023)	3 ♀
Osmia (Helicosmia) melanogaster Spinola, 1808	Proshchalykin et al. (2019); Proshchalykin and Maharramov (2020)	60 ♀, 24 ♂
Osmia (Helicosmia) niveata (Fabricius, 1804)	Proshchalykin and Maharramov (2020)	1 ♀, 1 ♂
Osmia (Helicosmia) saxatilis Warncke, 1988	Proshchalykin and Maharramov (2020)	1 ♀
Osmia (Helicosmia) signata Erichson, 1835	Proshchalykin et al. (2019); Proshchalykin and Maharramov (2020); Ascher and Pickering (2025)	2 ♀, 6 ♂
Osmia (Helicosmia) subcornuta Morawitz, 1875	Proshchalykin and Maharramov (2020)	3 ♀, 2 ♂
Osmia (Hemiosmia) difficilis Morawitz, 1875	Proshchalykin et al. (2019); Proshchalykin and Maharramov (2020); Ascher and Pickering (2025)	2 ♀, 1 ♂
Osmia (Hoplosmia) bidentata Morawitz, 1876	Maharramov et al. (2014); Proshchalykin and Maharramov (2020)	8 ♀, 13 ♂
Osmia (Hoplosmia) distinguenda (Tkalců, 1974)	Proshchalykin et al. (2019); Ascher and Pickering (2025)	1 ♀
Osmia (Hoplosmia) ligurica Morawitz, 1868	Proshchalykin and Maharramov (2020)	11 ♀, 1 ♂
Osmia (Hoplosmia) scutellaris Morawitz, 1868	Proshchalykin and Maharramov (2020)	3 ♀, 6 ♂
Osmia (Melanosmia) parietina Curtis, 1828	Maharramov et al. (2014)	–
Osmia (Metallinella) brevicornis (Fabricius, 1798)	Maharramov et al. (2014); Proshchalykin and Maharramov (2020)	35 ♀, 8 ♂
Osmia (Osmia) apicata Smith, 1853	Maharramov et al. (2014), as *O. macroglossa*; Proshchalykin and Maharramov (2020)	22 ♀, 57 ♂
Osmia (Osmia) bicornis globosa (Scopoli, 1763)	Maharramov et al. (2014), as *O. rufa*; Proshchalykin et al. (2019); Proshchalykin and Maharramov (2020)	4 ♀, 4 ♂
Osmia (Osmia) cerinthidis Morawitz, 1876	Maharramov et al. (2014); Proshchalykin and Maharramov (2020)	4 ♀, 4 ♂
Osmia (Osmia) cornuta quasirufa Peters, 1978	Maharramov et al. (2014), as *O. cornuta*; Proshchalykin et al. (2019)	4 ♀, 1 ♂
Osmia (Osmia) mustelina Gerstäcker, 1869	Proshchalykin et al. (2019); Ascher and Pickering (2025)	1 ♀, 1 ♂
Osmia (Osmia) nigrohirta Friese, 1899	Proshchalykin and Maharramov (2020)	19 ♀, 1 ♂
Osmia (Osmia) scheherazade Peters, 1978**	–	1 ♀
Osmia (Pyrosmia) amathusica Mavromoustakis, 1937	Maharramov et al. (2023)	1 ♀
Osmia (Pyrosmia) cephalotes longiceps Morawitz, 1876	Proshchalykin and Maharramov (2020)	15 ♀
Osmia (Pyrosmia) cyanoxantha Pérez, 1879	Proshchalykin and Maharramov (2020)	9 ♀
Osmia (Pyrosmia) dilaticornis Morawitz, 1875	Maharramov et al. (2023)	1 ♂
Osmia (Pyrosmia) forticornis van der Zanden, 1989	Proshchalykin and Maharramov (2020)	1 ♀
Osmia (Pyrosmia) nana Morawitz, 1874	Maharramov et al. (2014)	–
Osmia (Pyrosmia) versicolor Latreille, 1811	Proshchalykin et al. (2019); Ascher and Pickering (2025)	29 ♀, 4 ♂
Osmia (Pyrosmia) viridana Morawitz, 1873	Maharramov et al. (2023)	3 ♀, 2 ♂
Osmia (Tergosmia) rhodoensis (van der Zanden, 1983)**	–	3 ♀
Protosmia (Nanosmia) magnicapitis (Stanek, 1969)**	–	1 ♀
Protosmia (Protosmia) glutinosa (Giraud, 1871)	Maharramov et al. (2023)	3 ♀
**Tribe Anthidiini**		
Afranthidium (Mesanthidium) carduele (Morawitz, 1876)	Fateryga et al. (2020)	3 ♀, 7 ♂
Afranthidium (Mesanthidium) pusillum (Morawitz, 1894)	Fateryga et al. (2020)	1 ♀
Anthidiellum (Anthidiellum) strigatum (Panzer, 1805)	Warncke (1980); Maharramov et al. (2014); Fateryga et al. (2020)	1 ♀, 8 ♂
Anthidiellum (Anthidiellum) troodicum (Mavromoustakis, 1949)	Fateryga et al. (2020), as *A. breviusculum*	2 ♂
Anthidium (Anthidium) akanthurum Nadimi & Talebi, 2014, sp. resurr.**	Fateryga et al. (2020), as *A. gussakovskiji* (misidentified)	1 ♀, 2 ♂
Anthidium (Anthidium) cingulatum Latreille, 1809	Maharramov et al. (2014); Fateryga et al. (2020)	2 ♀, 6 ♂
Anthidium (Anthidium) dalmaticum Mocsáry, 1884	Fateryga et al. (2020)	9 ♀, 1 ♂
Anthidium (Anthidium) diadema Latreille, 1809	Maharramov et al. (2014); Fateryga et al. (2020)	1 ♀, 5 ♂
Anthidium (Anthidium) florentinum (Fabricius, 1775)	Maharramov et al. (2014); Fateryga et al. (2020)	11 ♀, 6 ♂
Anthidium (Anthidium) punctatum Latreille, 1809, s. l.	Fateryga et al. (2020)	5 ♀, 14 ♂
Anthidium (Anthidium) loti Perris, 1852	Maharramov et al. (2014); Fateryga et al. (2020)	3 ♀, 5 ♂
Anthidium (Anthidium) manicatum (Linnaeus, 1758)	Warncke (1980); Maharramov et al. (2014)	–
Anthidium (Anthidium) melanopygum Friese, 1917	Fateryga et al. (2020), as *A. spiniventre*	10 ♀, 11 ♂
Anthidium (Anthidium) taeniatum Latreille, 1809	Fateryga et al. (2020)	1 ♀, 4 ♂
Anthidium (Anthidium) wuestneii Mocsáry, 1887	Warncke (1980); Fateryga et al. (2020)	12 ♀, 16 ♂
Anthidium (Gulanthidium) rotundum Warncke, 1980	Fateryga et al. (2020)	7 ♀, 13 ♂
Anthidium (Proanthidium) oblongatum (Illiger, 1806)	Maharramov et al. (2014); Fateryga et al. (2020)	1 ♀
Anthidium (Turkanthidium) unicum Morawitz, 1875	Maharramov et al. (2023)	1 ♀
Eoanthidium (Eoanthidium) clypeare (Morawitz, 1874)	Maharramov et al. (2014); Ascher and Pickering (2025)	–
*Icteranthidium aequabile* (Morawitz, 1896)	Warncke (1980); Ascher and Pickering (2025)	–
*Icteranthidium cimbiciforme* (Smith, 1854)	Fateryga et al. (2020)	8 ♀, 7 ♂
*Icteranthidium croceum* (Morawitz, 1877)	Maharramov et al. (2014); Fateryga et al. (2020)	2 ♀
*Icteranthidium grohmanni* (Spinola, 1838)	Warncke (1980); Fateryga et al. (2020)	2 ♀, 2 ♂
*Icteranthidium limbiferum* (Morawitz, 1875)	Fateryga et al. (2020)	2 ♀
*Icteranthidium urfanum* (Warncke, 1980)	Fateryga et al. (2020)	1 ♀
Pseudoanthidium (Exanthidium) astafurovae Fateryga, Maharramov & Proshchalykin, sp. nov.	–	1 ♂
Pseudoanthidium (Exanthidium) eximium (Giraud, 1863)	Fateryga et al. (2020)	1 ♀, 1 ♂
Pseudoanthidium (Pseudoanthidium) melanurum (Klug, 1832)	Maharramov et al. (2014); Fateryga et al. (2020)	8 ♂
Pseudoanthidium (Pseudoanthidium) nanum (Mocsáry, 1880)	Fateryga et al. (2020)	2 ♂
Pseudoanthidium (Pseudoanthidium) reticulatum (Mocsáry, 1884)	Fateryga et al. (2020)	3 ♀, 4 ♂
Pseudoanthidium (Pseudoanthidium) rhombiferum (Friese, 1917)**	–	5 ♀, 3 ♂
Pseudoanthidium (Pseudoanthidium) stigmaticorne (Dours, 1873)*	–	1 ♀, 1 ♂
Pseudoanthidium (Pseudoanthidium) syriacum Kasparek, 2024**	–	1 ♀
Pseudoanthidium (Pseudoanthidium) tenellum (Mocsáry, 1880)	Fateryga et al. (2020)	1 ♂
Rhodanthidium (Asianthidium) aculeatum (Klug, 1832)	Fateryga et al. (2020)	1 ♀, 4 ♂
Rhodanthidium (Asianthidium) caturigense (Giraud, 1863)	Fateryga et al. (2020); Ascher and Pickering (2025)	1 ♀, 2 ♂
Rhodanthidium (Meganthidium) superbum (Radoszkowski, 1876)	Maharramov et al. (2023)	1 ♀
Rhodanthidium (Rhodanthidium) septemdentatum (Latreille, 1809)	Warncke (1980); Maharramov et al. (2014); Fateryga et al. (2020)	8 ♀, 10 ♂
Stelis (Protostelis) signata (Latreille, 1809)	Warncke (1992)	1 ♀, 2 ♂
Stelis (Pseudostelis) denticulata Friese, 1899	Maharramov et al. (2023)	1 ♂
Stelis (Pseudostelis) minima Schenck, 1861	Maharramov et al. (2023), as *S. minuta*	1 ♀
Stelis (Pseudostelis) minuta Lepeletier de Saint-Fargeau & Audinet-Serville, 1825	Warncke (1992); Maharramov et al. (2023)	1 ♂
Stelis (Stelidomorpha) nasuta (Latreille, 1809)*	–	1 ♀
Stelis (Stelis) breviuscula (Nylander, 1848)	Maharramov et al. (2023)	1 ♀, 2 ♂
Stelis (Stelis) odontopyga Noskiewicz, 1926	Maharramov et al. (2014)	–
Stelis (Stelis) phaeoptera (Kirby, 1802)*	–	1 ♀, 1 ♂
Stelis (Stelis) scutellaris Morawitz, 1893	Fateryga et al. (2020)	4 ♀
Stelis (Stelis) simillima Morawitz, 1876	Warncke (1992); Fateryga et al. (2020)	1 ♂
Trachusa (Archianthidium) pubescens (Morawitz, 1872)	Fateryga et al. (2020); Maharramov et al. (2023)	2 ♀, 1 ♂
Trachusa (Paraanthidium) anatolica Kasparek, 2020	Ascher and Pickering (2025)	–
Trachusa (Paraanthidium) heinzi Dubitzky, 2007	Fateryga et al. (2020)	1 ♂
Trachusa (Trachusa) byssina (Panzer, 1798)	Fateryga et al. (2020)	1 ♂
**Tribe Dioxyini**		
*Aglaoapis tridentata* (Nylander, 1848)	Maharramov et al. (2014)	–
*Metadioxys graeca* (Mavromoustakis, 1963)**	Maharramov et al. (2021), as *M. formosus* (misidentified)	3 ♀
**Tribe Megachilini**		
Coelioxys (Allocoelioxys) acanthura (Illiger, 1806)	Maharramov et al. (2021)	1 ♀
Coelioxys (Allocoelioxys) afer Lepeletier de Saint-Fargeau, 1841	Maharramov et al. (2021)	2 ♀, 4 ♂
Coelioxys (Allocoelioxys) argenteus Lepeletier de Saint-Fargeau, 1841**	–	1 ♂
Coelioxys (Allocoelioxys) castaneus Morawitz, 1886	Morawitz (1886); Schwarz and Gusenleitner (2003); Ascher and Pickering (2025)	1 ♀
Coelioxys (Allocoelioxys) caudatus Spinola, 1838	Maharramov et al. (2023)	1 ♂
Coelioxys (Allocoelioxys) echinatus Förster, 1853	Maharramov et al. (2014, 2021)	1 ♀, 7 ♂
Coelioxys (Allocoelioxys) haemorrhoa Förster, 1853	Maharramov et al. (2021)	2 ♂
Coelioxys (Allocoelioxys) obtusus Pérez, 1884**	–	1 ♂
Coelioxys (Coelioxys) quadridentatus (Linnaeus, 1758)**	–	2 ♀
Coelioxys (Melissoctonia) conoideus (Illiger, 1806)	Maharramov et al. (2023)	1 ♀
Coelioxys (Paracoelioxys) elongatus Lepeletier de Saint-Fargeau, 1841	Maharramov et al. (2014)	–
Coelioxys (Rozeniana) aurolimbatus Förster, 1853	Maharramov et al. (2021)	1 ♀, 2 ♂
Coelioxys (Rozeniana) rufescens Lepeletier de Saint-Fargeau & Audinet-Serville, 1825	Maharramov et al. (2014)	1 ♀
Megachile (Chalicodoma) albocristata Smith, 1853	Maharramov et al. (2021)	4 ♀, 4 ♂
Megachile (Chalicodoma) albonotata Radoszkowski, 1886	Maharramov et al. (2021)	21 ♀, 4 ♂
Megachile (Chalicodoma) montenegrensis Dours, 1873	Maharramov et al. (2014), as *M. ponticum*; Maharramov et al. (2021)	13 ♀, 6 ♂
Megachile (Chalicodoma) parietina (Geoffroy, 1785)	Maharramov et al. (2014, 2021)	7 ♀, 2 ♂
Megachile (Chalicodoma) pyrenaica (Lepeletier de Saint-Fargeau, 1841)	Maharramov et al. (2014, 2021)	17 ♀, 4 ♂
Megachile (Creightonella) albisecta (Klug, 1817)	Maharramov et al. (2014, 2021)	19 ♀, 14 ♂
Megachile (Creightonella) doriae Magretti, 1890	Maharramov et al. (2021)	1 ♀, 4 ♂
Megachile (Eutricharaea) anatolica Rebmann, 1968**	–	1 ♀, 2 ♂
Megachile (Eutricharaea) apicalis Spinola, 1808	Maharramov et al. (2014, 2021)	27 ♀, 23 ♂
Megachile (Eutricharaea) argentata (Fabricius, 1793)	Maharramov et al. (2021), as *M. pilidens*	16 ♀, 6 ♂
Megachile (Eutricharaea) burdigalensis Benoist, 1940	Maharramov et al. (2021)	4 ♀, 2 ♂
Megachile (Eutricharaea) deceptoria Pérez, 1890	Maharramov et al. (2014, 2021)	9 ♀, 13 ♂
Megachile (Eutricharaea) giraudi Gerstäcker, 1869	Maharramov et al. (2021)	2 ♂
Megachile (Eutricharaea) leachella Curtis, 1828	Maharramov et al. (2021)	36 ♀, 54 ♂
Megachile (Eutricharaea) leucomalla Gerstäcker, 1869	Maharramov et al. (2021)	6 ♀, 5 ♂
Megachile (Eutricharaea) marginata Smith, 1853	Maharramov et al. (2021)	8 ♀, 6 ♂
Megachile (Eutricharaea) melanogaster Eversmann, 1852	Maharramov et al. (2021)	2 ♀, 2 ♂
Megachile (Eutricharaea) rotundata (Fabricius, 1787)	Maharramov et al. (2014, 2021)	19 ♀, 21 ♂
Megachile (Eutricharaea) semicircularis auct. nec van der Zanden, 1996	Maharramov et al. (2021)	31 ♀, 7 ♂
Megachile (Megachile) centuncularis (Linnaeus, 1758)	Maharramov et al. (2014, 2021)	3 ♀, 2 ♂
Megachile (Megachile) genalis Morawitz, 1880**	–	4 ♀
Megachile (Megachile) melanopyga Costa, 1863	Maharramov et al. (2014, 2021)	2 ♀, 1 ♂
Megachile (Megachile) octosignata Nylander, 1852	Maharramov et al. (2021)	1 ♀, 1 ♂
Megachile (Megachile) pilicrus Morawitz, 1877	Maharramov et al. (2021)	15 ♀, 3 ♂
Megachile (Megachile) versicolor Smith, 1844	Maharramov et al. (2014, 2021)	2 ♀
Megachile (Pseudomegachile) ericetorum Lepeletier de Saint-Fargeau, 1841	Maharramov et al. (2021)	8 ♀, 11 ♂
Megachile (Pseudomegachile) flavipes Spinola, 1838	Maharramov et al. (2014, 2021)	12 ♀, 22 ♂
Megachile (Pseudomegachile) saussurei Radoszkowski, 1874	Maharramov et al. (2014)	12 ♀, 1 ♂
Megachile (Pseudomegachile) tecta Radoszkowski, 1888	Maharramov et al. (2021)	24 ♀, 9 ♂
Megachile (Xanthosarus) circumcincta (Kirby, 1802)	Maharramov et al. (2014, 2021)	6 ♀, 12 ♂
Megachile (Xanthosarus) lagopoda (Linnaeus, 1761)	Maharramov et al. (2014, 2021)	1 ♂
Megachile (Xanthosarus) maritima (Kirby, 1802)	Maharramov et al. (2014, 2021)	16 ♀, 5 ♂
Megachile (Xanthosarus) willughbiella (Kirby, 1802)	Maharramov et al. (2021)	2 ♀

### ﻿New species records for Azerbaijan

#### ﻿ Chelostoma (Foveosmia) foveolatum

Taxon classificationAnimaliaHymenopteraMegachilidae

(Morawitz, 1868)

F6C1C008-C9AE-56C0-AE50-8B8AE4B6B806

##### Material examined.

**Azerbaijan. Nakhchivan Autonomous Republic** • Ordubad, Mazra, 39°03'25"N, 45°55'30"E, 1615 m, 16.VI.2024, 4 ♀, 2 ♂, leg. M. Proshchalykin, M. Maharramov [FSCV].

##### Distribution.

Western, Southern, and Eastern Europe, Russia (European part), Georgia, Azerbaijan, Turkey, Lebanon.

#### ﻿ Heriades (Michenerella) punctulifera

Taxon classificationAnimaliaHymenopteraMegachilidae

Schletterer, 1889

DCBC5436-351A-5FA4-88C8-EC6127134175

##### Material examined.

**Azerbaijan. Nakhchivan Autonomous Republic** • Nakhchivan, 39°12'24"N, 45°43'49"E, 830 m, 4.VIII.2023, 1 ♀, leg. M. Maharramov [CAFK]; • Julfa, Daridagh-2, 39°03'58"N, 45°37'35"E, 1100 m, 30.V.2025, 1 ♀, leg. M. Proshchalykin, M. Maharramov [CAFK].

##### Distribution.

Southern and Eastern Europe, Azerbaijan, Turkey, Cyprus, Syria, Lebanon, Israel and Palestine.

#### ﻿ Hoplitis (Alcidamea) brachypogon

Taxon classificationAnimaliaHymenopteraMegachilidae

(Pérez, 1879)

88DB4314-C49E-5DF6-AD01-9255DF98E67F

##### Material examined.

**Azerbaijan. Nakhchivan Autonomous Republic** • Shakhbuz, Kolani, 39°26'51"N, 45°39'13"E, 1330 m, 21.V.2025, 1 ♂, leg. M. Proshchalykin, M. Maharramov [CAFK].

##### Distribution.

Western, Southern, and Eastern Europe, Azerbaijan, Turkey, Syria, Israel and Palestine, Iran.

#### ﻿ Hoplitis (Alcidamea) campanularis

Taxon classificationAnimaliaHymenopteraMegachilidae

(Morawitz, 1877)

933E11CF-9DB3-55AB-B067-1E4E84CB3796

##### Material examined.

**Azerbaijan. Nakhchivan Autonomous Republic** • Shakhbuz, Kolani, 39°26'51"N, 45°39'13"E, 1330 m, 28.V.2025, 2 ♀, leg. M. Proshchalykin, M. Maharramov [FSCV].

##### Distribution.

Southern and Eastern Europe, Northern Africa, Russia (European part), Georgia, Azerbaijan, Turkey, Lebanon, Israel and Palestine.

#### ﻿ Hoplitis (Anthocopa) mocsaryi

Taxon classificationAnimaliaHymenopteraMegachilidae

(Friese, 1895)

6850CEAF-F318-50D6-9DFC-0B20EB8E681D

##### Material examined.

**Azerbaijan. Nakhchivan Autonomous Republic** • Shakhbuz, Kulus-2, 39°21'51"N, 45°37'38"E, 1400 m, 28.V.2025, 1 ♀, leg. M. Proshchalykin, M. Maharramov [FSCV].

##### Distribution.

Western, Southern, and Eastern Europe, Russia (European part), Armenia, Azerbaijan, Turkey, Israel and Palestine, Iran.

#### ﻿ Osmia (Helicosmia) breviata

Taxon classificationAnimaliaHymenopteraMegachilidae

Warncke, 1988

5178F023-E194-55AD-8383-D642021F2FF7

##### Material examined.

**Azerbaijan. Nakhchivan Autonomous Republic** • Shakhbuz, Kechili, 39°22'N, 45°43'E, 1800 m, 12.VII.2019, 1 ♀, leg. M. Maharramov [CAFK].

##### Distribution.

Southern Europe, Russia (European part), Azerbaijan, Turkey, Lebanon, Israel and Palestine, Iran.

#### ﻿ Osmia (Helicosmia) clypearisacuta

Taxon classificationAnimaliaHymenopteraMegachilidae

Warncke, 1988

D9920733-367A-551C-AF7B-0DEF8B72FECC

##### Material examined.

**Azerbaijan. Nakhchivan Autonomous Republic** • Sharur, Shahbulag, 39°38'57"N, 45°08'12"E, 1210 m, 26.V.2025, 3 ♀, leg. M. Proshchalykin, M. Maharramov [2 ♀, CAFK; 1 ♀, FSCV]; • Julfa, Daridagh-2, 39°03'58"N, 45°37'35"E, 1100 m, 29.V.2025, 1 ♀, leg. M. Proshchalykin, M. Maharramov [FSCV].

##### Distribution.

Azerbaijan, Turkey, Syria, Jordan, Lebanon, Israel and Palestine, Iraq.

#### ﻿ Osmia (Osmia) scheherazade

Taxon classificationAnimaliaHymenopteraMegachilidae

Peters, 1978

EEE18DF4-2955-5741-9D69-37587B8E491A

##### Material examined.

**Azerbaijan. Nakhchivan Autonomous Republic** • Shakhbuz, Badamli, 39°28'05"N, 45°33'00"E, 1290 m, 21.V.2025, 1 ♀, leg. M. Proshchalykin, M. Maharramov [FSCV].

##### Distribution.

Russia (European part), Azerbaijan, Turkey, Iran.

#### ﻿ Osmia (Tergosmia) rhodoensis

Taxon classificationAnimaliaHymenopteraMegachilidae

(van der Zanden, 1983)

E96E63A2-89C9-58E8-96A9-E00458E02A86

##### Material examined.

**Azerbaijan. Nakhchivan Autonomous Republic** • Shakhbuz, Kulus, 39°21'56"N, 45°40'54"E, 1620 m, 23.V.2025, 1 ♀, leg. M. Proshchalykin, M. Maharramov [FSCV]; • ibid., 28.V.2025, 2 ♀, leg. M. Proshchalykin, M. Maharramov [CAFK].

##### Distribution.

Southern Europe, Armenia, Azerbaijan, Turkey, Syria, Jordan, Lebanon, Israel and Palestine, Iran.

#### ﻿ Protosmia (Nanosmia) magnicapitis

Taxon classificationAnimaliaHymenopteraMegachilidae

(Stanek, 1969)

FEDFAEA4-E2CA-5FFB-8CE9-3422E99CD6B7

##### Material examined.

**Azerbaijan. Nakhchivan Autonomous Republic** • Julfa, Daridagh-2, 1100 m, 39°03'58"N, 45°37'35"E, 20.VI.2024, 1 ♀, leg. M. Proshchalykin, M. Maharramov [ETHZ].

##### Distribution.

Azerbaijan, Turkey.

#### ﻿ Anthidium (Anthidium) akanthurum

Taxon classificationAnimaliaHymenopteraMegachilidae

Nadimi & Talebi, 2014, sp. resurr.

3D33B385-CE3B-52E3-9AD9-0EFFF90CEE56

Fig. 2

##### Description of male (hitherto unknown).

Length 8 mm. ***Head***: clypeus 1.35× as long as broad, distinctly convex, pale yellow, densely punctured, with broad polished longitudinal line in middle; apical margin straight, with narrow translucent lamella; paraocular area densely punctured, to level of insertion of antenna pale yellow; clypeus, paraocular area, and frons with dense white hairs about as long as width of mandible; gena and occiput with nearly complete fused yellow band, narrowly interrupted only in middle of occiput; frons and vertex black; vertex and occiput with yellowish hairs longer than those on clypeus, paraocular area, and frons; distance between lateral ocellus and occiputal margin about 2.3× diameter of lateral ocellus; mandible pale yellow, tridentate, teeth black; scape black dorsally and yellow ventrally, pedicel mostly black, flagellum brown dorsally and light brown ventrally. ***Mesosoma***: scutum densely punctured, moderately shining, with greyish-yellow hairs as those on vertex and occiput, not hiding surface, with L-shaped yellow anterolateral stripe at each side; mesepisternum to propodeum densely covered with white hairs as those on clypeus, paraocular area, and frons; tegula pale yellow, disk brown in middle; scutellum slightly produced over propodeum, densely punctured, moderately shining, with greyish-yellow hairs, black with apical yellow band interrupted medially; apical margin of scutellum rounded, very slightly emarginate in middle, not lamellate; axilla not united with scutellum, black with posterolateral yellow spot, laterally rounded; pronotal lobe pale yellow, otherwise mesosoma black; second recurrent vein intersticial with apex of second transverse cubital vein; basal vein originating considerably basad of transverse median vein; legs mostly yellow with ferruginous, densely pubescent similarly to mesepisternum to propodeum; outer face of hind tibia rounded; hind spurs pale yellow. ***Metasoma***: terga 1–6 rather dull, with fine dense punctures on discs, interspaces reaching at most puncture diameter; tergal discs reddish-brown basally and with complete yellow bands apically; depression with much denser and finer punctures, completely dull, brown and translucent; terga with few short erect whitish hairs and apical bands of appressed white hairs, more distinct on terga 3 and 4 and present only laterally on terga 1 and 2; tergum 6 with hooked lateral projections; tergum 7 nearly completely yellow, apically tridentate but mid tooth very small, with apical translucent margin; sterna very shallowly and indistinctly punctured, rather shining, mostly brown with little black and yellow areas; apical margins of sterna 1–5 with bands of appressed white hairs; sternum 6 medially with broad polished longitudinal line lacking pilosity, laterally producing as long strong acute tooth, apically with long, anchor-shaped extension; sternum 7 deeply divided medially on two apically rounded lobes; sternum 8 with rounded postero-lateral lobes and narrow hairy apical projection; genitalia typical of the genus *Anthidium* Fabricius, 1804, with penis valves broadly separated and connected subapically; penis valve apically curved and tapering; gonostylus hairy, just singly enlarged towards apex and apically rounded.

##### Material examined.

**Azerbaijan. Nakhchivan Autonomous Republic** • Julfa, 5 km N Dize, 39°03'N, 45°45'E, 965 m, 20.VI.2019, 1 ♂, leg. M. Proshchalykin, Kh. Aliyev, M. Maharramov [CMKH]; • Ordubad, Bilav, 39°02'43"N, 45°49'07"E, 1050 m, 22.V.2025, 1 ♀, leg. M. Proshchalykin, M. Maharramov [ZISP]; • ibid., 27.V.2025, 1 ♂, leg. M. Proshchalykin, M. Maharramov [ZISP].

##### Distribution.

Azerbaijan, Iran.

##### Remarks.

**Figure 2.**
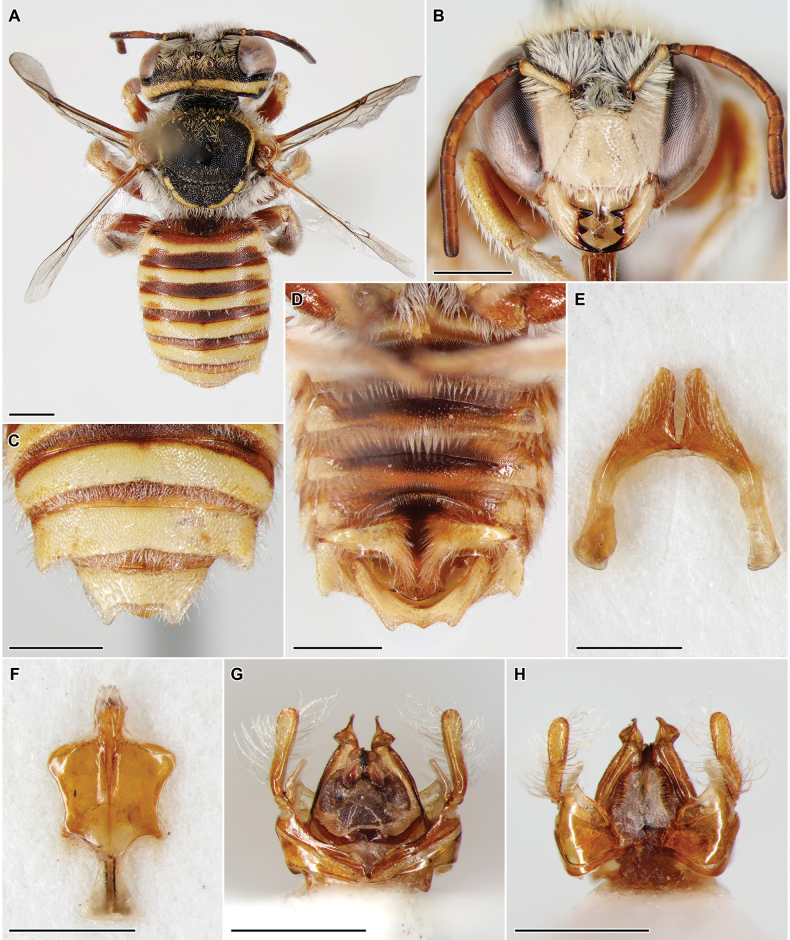
*Anthidium akanthurum* Nadimi & Talebi, 2014, sp. resurr., ♂, Azerbaijan. A. Habitus in dorsal view; B. Head in frontal view; C. Metasomal terga 4–6; D. Metasoma in ventral view; E. Sternum 7; F. Sternum 8; G. Genitalia in dorsal view; H. Genitalia in ventral view. Scale bars: 1 mm.

Fateryga et al. (2020) erroneously synonymized this species with Anthidium (Anthidium) gussakovskiji Mavromoustakis, 1939 and reported the latter taxon from Azerbaijan. *Anthidium akanthurum* was described based on females only (Nadimi et al. 2014), while a single male was available to Fateryga et al. (2020). Later, Kasparek (2022a, 2022b) published images of the male *A. gussakovskiji* and Maharramov et al. (2023) found the specimen examined by Fateryga et al. (2020) different, definitely belonging to a distinct species. Therefore, *A. gussakovskiji* was excluded from the fauna of Azerbaijan (Maharramov et al. 2023). Newly collected material of both sexes allowed us to ascertain that “*A. gussakovskiji*” sensu Fateryga et al. (2020) indeed belonged to *A. akanthurum*, despite that it was not conspecific to *A. gussakovskiji*. Therefore, the hitherto unknown male of *A. akanthurum* is formally described here and the species is resurrected from the incorrect synonymy with *A. gussakovskiji*. The main difference between males of these two species is in sternum 6, which lateral projection is directed rather posteriorly in *A. gussakovskiji* (fig. 4C in Kasparek 2022a and fig. C on p. 104 in Kasparek 2022b) but strongly laterally in *A. akanthurum* (Fig. 2D).

#### ﻿ Pseudoanthidium (Exanthidium) astafurovae

Taxon classificationAnimaliaHymenopteraMegachilidae

Fateryga, Maharramov & Proshchalykin sp. nov.

488683F9-FE32-5F09-8DAD-74B1BF0D2E5A

https://zoobank.org/FA0A1451-3A23-4D29-8168-4E31E85DF191

Fig. 3

##### Diagnosis.

**Figure 3.**
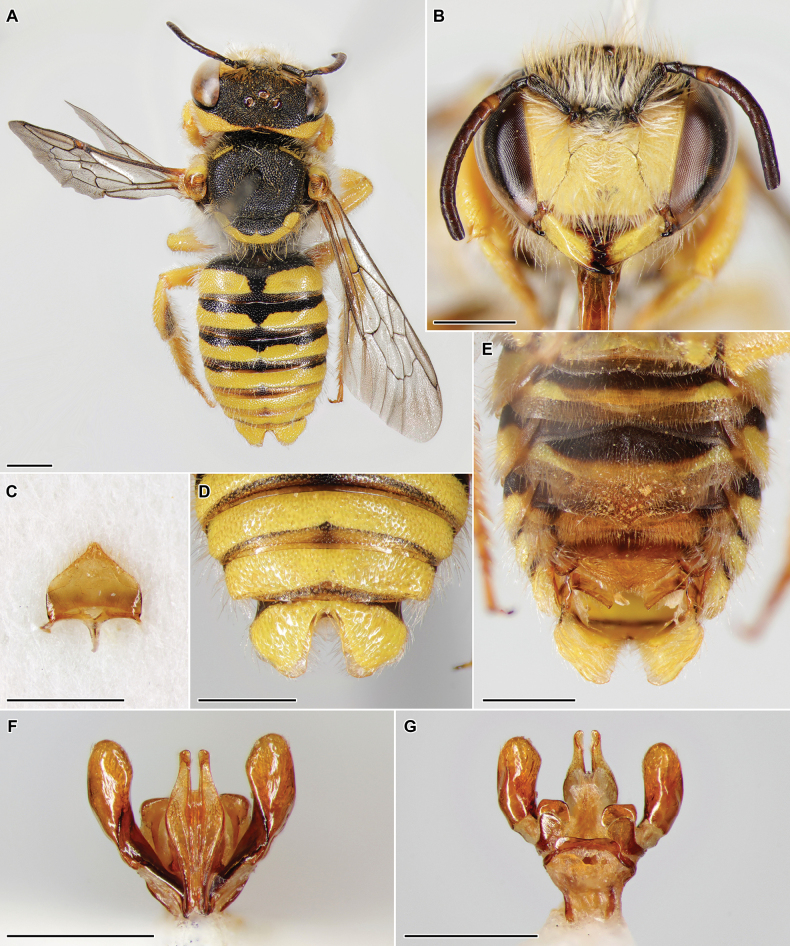
*Pseudoanthidium astafurovae* Fateryga, Maharramov & Proshchalykin, sp. nov., ♂, holotype. A. Habitus in dorsal view; B. Head in frontal view; C. Sternum 8; D. Metasomal terga 4–6; E. Metasoma in ventral view; F. Genitalia in dorsal view; G. Genitalia in ventral view. Scale bars: 1 mm.

Among the species of the subgenus Exanthidium Pasteels, 1969 (Kasparek 2021), the male of *P. astafurovae* is unequivocally characterised by the following combination of characters: i) tergum 6 laterally bulging, without hooked or spiniform protrusion (Fig. 3D); ii) emargination of tergum 7 little widening in apical third, with rather pointed base (emargination V-shaped) (Fig. 3D); iii) emargination of tergum 7 with translucent margin (Fig. 3D); iv) posterior margin of scutellum rather rounded in dorsal view (Fig. 3A); v) apical margin of sternum 2 truncate, without medial protrusion (Fig. 3E); vi) yellow preoccipital band reaching mandibular base. The new species is probably closely related to *Pseudoanthidium eximium* (Giraud, 1863), another species of the subgenus Exanthidium occurring in Azerbaijan, but the latter has a hooked lateral protrusion of tergum 6, rather straight posterior margin of scutellum in dorsal view, and a remarkably paler and less rich yellow pattern of the body (figs 6, 7 in Kasparek 2021). Besides this, *P. astafurovae* is distinct from *P. eximium* by a slenderer penis valve (especially its apical part) with much shorter hairs (Fig. 3F, G vs fig. 3G–I in Kasparek 2021) and distinctly pointed (not rather rounded) apical margins of sternum 7 (Fig. 3E), as well as a distinctly pointed apical margin of sternum 8 (Fig. 3C).

##### Description.

**Male.** Length 8 mm. ***Head***: clypeus 1.4× as long as broad, yellow, densely and shallowly punctured, without longitudinal line in middle; narrow apical margin brownish and weakly crenulate; paraocular area densely punctured, to level of insertion of antenna and slightly above yellow; clypeus and paraocular area with dense white hairs as long as width of mandible; gena and occiput with complete fused yellow band, much narrowing in middle of occiput; frons and vertex mostly black, with yellowish hairs longer and sparser than those on clypeus and paraocular area; distance between lateral ocellus and occiputal margin 2× as diameter of lateral ocellus; mandible yellow, tridentate, teeth black; antenna mostly black except light brown ventral side of article 4 and brown ventral side of articles 5–13. ***Mesosoma***: scutum densely punctured, moderately shining, with greyish-yellow short hairs (shorter than those on frons and vertex) not hiding surface, with transverse anterolateral yellow stripe at each side; mesepisternum to propodeum densely covered with white hairs as those on clypeus and paraocular area; tegula yellow, disk brown in middle; scutellum slightly produced over propodeum, densely punctured, moderately shining, with greyish-yellow short hairs, black in anterior half and yellow in posterior half; apical margin of scutellum rounded, distinctly lamellate laterally; axilla not united with scutellum, yellow except small inner black part, laterally rather rounded; pronotal lobe yellow, otherwise mesosoma black; second recurrent vein distad to apex of second transverse cubital vein; basal vein originating considerably basad of transverse median vein; legs yellow except partially black coxae and trochanters, rather sparsely pubescent; outer face of hind tibia rounded; hind spurs pale yellow. ***Metasoma***: terga 1–6 distinctly shining, with fine punctures on discs, interspaces sometimes reaching more than puncture diameter in middle, narrower laterally, polished; tergal discs black basally and with yellow bands apically, interrupted on terga 1 and 2; depression with somewhat denser punctures, brown and translucent; terga with short sparse hairs; tergum 6 without lateral projections; tergum 7 nearly completely yellow, deeply V-shapely emarginate, with narrow translucent margin; sterna punctured finely and densely than targa; sterna 1–5 mostly black basally and yellow apically, sterna 6–8 mostly brown and translucent; apical margins of sterna with regular rows of dense specialized hairs, especially long and hiding structure on sternum 3; sternum 6 medially with rounded area lacking pilosity, apically rounded, with small unclear medioapical protrusion; sternum 7 with distinctly pointed apical margins; sternum 8 helmet-shaped; genitalia with comparatively slender penis valve, covered dorsomedially with short hairs, and enlarged towards apex and apically rounded gonostylus, nearly without hairs.

**Female.** Unknown.

##### Material examined.

***Holotype*: Azerbaijan. Nakhchivan Autonomous Republic** • Ordubad, Kotam, 38°53'25"N, 46°03'14"E, 700 m, 30.V.2025, 1 ♂, leg. M. Proshchalykin, M. Maharramov [ZISP].

##### Etymology.

The new species is named after our colleague Yulia V. Astafurova (ZISP), in recognition of her great contribution to the taxonomy of the bees of the Caucasus (including the bees of Azerbaijan).

##### Distribution.

Azerbaijan.

#### ﻿ Pseudoanthidium (Pseudoanthidium) rhombiferum

Taxon classificationAnimaliaHymenopteraMegachilidae

(Friese, 1917)

3E782128-9E46-51F1-9358-4B332242747D

##### Material examined.

**Azerbaijan. Nakhchivan Autonomous Republic** • Julfa, Daridagh-2, 39°03'58"N, 45°37'35"E, 1100 m, 18–19.V.2025, 1 ♂, leg. M. Proshchalykin, M. Maharramov [CAFK]; • ibid., 24.V.2025, 4 ♀, 1 ♂, leg. M. Proshchalykin, M. Maharramov [2 ♀, 1 ♂, CAFK; 2 ♀, FSCV]; • ibid., 29.V.2025, 1 ♂, leg. M. Proshchalykin, M. Maharramov [ZISP]; • Ordubad, Bilav, 39°02'43"N, 45°49'07"E, 1050 m, 29.V.2025, 1 ♀, leg. M. Proshchalykin, M. Maharramov [ZISP].

##### Distribution.

Azerbaijan, Turkey, Syria, Lebanon, Israel and Palestine, ?Afghanistan, ?Turkmenistan.

##### Remarks.

All examined female specimens from Azerbaijan have completely pale-yellow clypeus and paraocular area, while these parts of the head should be black in *P. rhombiferum* according to Kasparek (2024). However, both sexes of this species otherwise fit the characters of *P. rhombiferum*.

#### ﻿ Pseudoanthidium (Pseudoanthidium) syriacum

Taxon classificationAnimaliaHymenopteraMegachilidae

Kasparek, 2024

95646C2E-74C4-5F43-BC32-09FE6EDE5C6D

##### Material examined.

**Azerbaijan. Nakhchivan Autonomous Republic** • Julfa, Daridagh-2, 1100 m, 39°03'58"N, 45°37'35"E, 22.VI.2024, 1 ♀, leg. M. Proshchalykin, M. Maharramov [ZISP].

##### Distribution.

Azerbaijan, Syria.

#### ﻿ Metadioxys graeca

Taxon classificationAnimaliaHymenopteraMegachilidae

(Mavromoustakis, 1963)

4CA458D0-E274-56B0-93A4-CBCA96E41E63

##### Material examined.

**Azerbaijan. Nakhchivan Autonomous Republic** • Kengerli, Chalkhangala, Gizmizidash, 25.VI.2020, 3 ♀, leg. M. Maharramov [1 ♀, CAFK; 2 ♀, FSCV].

##### Distribution.

Southern Europe, North Africa, Azerbaijan, Turkey, Israel and Palestine.

##### Remarks.

The studied specimens were misidentified by Maharramov et al. (2021) as *Metadioxys formosus* (Morawitz, 1875). Therefore, the latter species should be excluded from the fauna of Azerbaijan.

#### ﻿ Coelioxys (Allocoelioxys) argenteus

Taxon classificationAnimaliaHymenopteraMegachilidae

Lepeletier de Saint-Fargeau, 1841

DB051BCD-3837-5BFA-894C-540021E6A4E3

##### Material examined.

**Azerbaijan. Nakhchivan Autonomous Republic** • Nakhchivan, 39°12'24"N, 45°43'49"E, 830 m, 4.VIII.2023, 1 ♂, leg. M. Maharramov [FSCV].

##### Distribution.

Western, Southern, and Eastern Europe, North Africa, Russia (European part), Azerbaijan, Turkey, Cyprus, Syria, Jordan, Lebanon, Israel and Palestine, Iran, Turkmenistan, Tajikistan, Uzbekistan, Kyrgyzstan, Kazakhstan, China.

#### ﻿ Coelioxys (Allocoelioxys) obtusus

Taxon classificationAnimaliaHymenopteraMegachilidae

Pérez, 1884

D7D2DA4E-EFDE-5F74-910F-AA3AA5653D15

##### Material examined.

**Azerbaijan. Nakhchivan Autonomous Republic** • Babek, Shikhmakhmud, 39°15'55"N, 45°25'33"E, 940 m, 7.VIII.2023, 1 ♂, M. Maharramov [FSCV].

##### Distribution.

Western, Southern, and Eastern Europe, North Africa, Russia (European part), Azerbaijan, Turkey, Iraq, Iran, Turkmenistan.

#### ﻿ Coelioxys (Coelioxys) quadridentatus

Taxon classificationAnimaliaHymenopteraMegachilidae

(Linnaeus, 1758)

A3152C1B-C447-5DA1-B41B-5117E908BB11

##### Material examined.

**Azerbaijan. Nakhchivan Autonomous Republic** • Shakhbuz, Kulus, 39°21'56"N, 45°40'54"E, 1620 m, 23.V.2025, 2 ♀, leg. M. Proshchalykin, M. Maharramov [FSCV].

##### Distribution.

Western, Northern, Southern, and Eastern Europe, Russia (European part, Urals, Western Siberia, Eastern Siberia, Far East), Azerbaijan, Turkey, Iran, China.

#### ﻿ Megachile (Eutricharaea) anatolica

Taxon classificationAnimaliaHymenopteraMegachilidae

Rebmann, 1968

7CEA9DD8-1FA2-5839-8798-AE3EBBADD16B

##### Material examined.

**Azerbaijan. Nakhchivan Autonomous Republic** • Ordubad, Kotam, 38°53'25"N, 46°03'14"E, 700 m, 19.V.2025, 2 ♂, leg. M. Proshchalykin, M. Maharramov [FSCV]; • Ordubad, Bilav, 39°02'43"N, 45°49'07"E, 1050 m, 22.V.2025, 1 ♀, leg. M. Proshchalykin, M. Maharramov [FSCV].

##### Distribution.

Southern Europe, Russia (European part), Azerbaijan, Turkey, Cyprus, Jordan, Lebanon, Israel and Palestine, Iran.

#### ﻿ Megachile (Megachile) genalis

Taxon classificationAnimaliaHymenopteraMegachilidae

Morawitz, 1880

73D86E8D-0D17-5CDD-BD1B-2DE37C34EB84

##### Material examined.

**Azerbaijan. Nakhchivan Autonomous Republic** • Sharur, Shahbulag, 39°38'57"N, 45°08'12"E, 1210 m, 26.V.2025, 4 ♀, leg. M. Proshchalykin, M. Maharramov [1 ♀, CAFK; 3 ♀, FSCV].

##### Distribution.

Western, Northern, Southern, and Eastern Europe, Russia (European part, Urals, Western Siberia, Far East), Azerbaijan, Turkey, Tajikistan, Kyrgyzstan, Kazakhstan, Mongolia, China, Japan.

## ﻿Discussion

Maharramov et al. (2023) calculated that 174 species of megachilid bees were known from Azerbaijan and 160 of them were known from the Nakhchivan Autonomous Republic according to Maharramov et al. (2014, 2021, 2023), Proshchalykin et al. (2019), Fateryga et al. (2020), and Proshchalykin and Maharramov (2020). However, four species were overlooked in these calculations. Stelis (Pseudostelis) minima Schenck, 1861 was reported from the Nakhchivan AR by Maharramov et al. (2023) in remarks to S. (P.) minuta Lepeletier de Saint-Fargeau & Audinet-Serville, 1825 and Hoplitis (Hoplitis) astragali Fateryga, Müller & Proshchalykin, 2023 was described from Dagestan (Russia) with one paratype from the Nakhchivan AR (Fateryga et al. 2023). Megachile (Chalicodoma) monstrifica Morawitz, 1877 was described from the main territory of Azerbaijan (Morawitz 1877; Aliyev et al. 2018), and Pseudoanthidium (Pseudoanthidium) stigmaticorne (Dours, 1873) was reported from this country by Litman et al. (2021). In the present contribution, we have described a new species and reported 19 species new to Azerbaijan. In addition, three species previously known from Azerbaijan are reported as new to the Nakhchivan AR. At the same time, one species (*Metadioxys formosus*) is excluded from the fauna of Azerbaijan due to its incorrect previous identification by Maharramov et al. (2021). Another species that should be excluded from the fauna of Azerbaijan is Coelioxys (Allocoelioxys) sogdianus Morawitz, 1875 reported by Ascher and Pickering (2025) and mentioned also by Maharramov et al. (2021); its record was erroneous and based on a confusion with C. (A.) castaneus Morawitz, 1886, the only species of megachilid bees described from the Nakhchivan AR until recently (Schwarz and Gusenleitner 2003).

Thus, 184 species are currently known from the Nakhchivan AR. A total of 171 species were identified among the material collected in the autonomy; these species are undoubtedly present in the regional fauna. At the same time, 13 species are known from the Nakhchivan AR on the base of literature records only; the presence of these species in the autonomy is unconfirmed and requiring further verification. A total of 196 species of megachilid bees are currently known from Azerbaijan as a whole. Thus, just 12 species reported from this country were not recorded in the Nakhchivan AR to date. These species are Hoplitis (Alcidamea) caucasica (Friese, 1920), H. (A.) curvipes (Morawitz, 1871), H. (A.) verruciventris (Morawitz, 1886), H. (Anthocopa) agis (Benoist, 1929), H. (Hoplitis) carinata (Stanek, 1969), Osmia (Pyrosmia) hellados van der Zanden, 1984, Anthidium (Proanthidium) trispinosum Friese, 1917, A. (P.) undulatum Dours, 1873, A. (P.) venustum Morawitz, 1877, M. (Ch.) alborufa Friese, 1911, M. (Ch.) monstrifica, and M. (Pseudomegachile) cinnamomea Alfken, 1926 (Aliyev et al. 2018; Fateryga et al. 2020; Proshchalykin and Maharramov 2020; Maharramov et al. 2021; Ascher and Pickering 2025). Therefore, we can conclude that the main territory of Azerbaijan is still poorly explored. A similar conclusion was recently made during a study of the pollen wasps (Fateryga et al. 2025). Further collecting efforts may surely reveal the existence of more species in this region.

## Supplementary Material

XML Treatment for Chelostoma (Foveosmia) foveolatum

XML Treatment for Heriades (Michenerella) punctulifera

XML Treatment for Hoplitis (Alcidamea) brachypogon

XML Treatment for Hoplitis (Alcidamea) campanularis

XML Treatment for Hoplitis (Anthocopa) mocsaryi

XML Treatment for Osmia (Helicosmia) breviata

XML Treatment for Osmia (Helicosmia) clypearisacuta

XML Treatment for Osmia (Osmia) scheherazade

XML Treatment for Osmia (Tergosmia) rhodoensis

XML Treatment for Protosmia (Nanosmia) magnicapitis

XML Treatment for Anthidium (Anthidium) akanthurum

XML Treatment for Pseudoanthidium (Exanthidium) astafurovae

XML Treatment for Pseudoanthidium (Pseudoanthidium) rhombiferum

XML Treatment for Pseudoanthidium (Pseudoanthidium) syriacum

XML Treatment for Metadioxys graeca

XML Treatment for Coelioxys (Allocoelioxys) argenteus

XML Treatment for Coelioxys (Allocoelioxys) obtusus

XML Treatment for Coelioxys (Coelioxys) quadridentatus

XML Treatment for Megachile (Eutricharaea) anatolica

XML Treatment for Megachile (Megachile) genalis

## ﻿Additional information

### Use of AI

No use of AI was reported.

### Author ORCIDs

Alexander V. Fateryga https://orcid.org/0000-0002-5346-3477

Mahir M. Maharramov https://orcid.org/0000-0002-4130-7071

Maksim Yu. Proshchalykin https://orcid.org/0000-0001-7870-8226

### Data availability

All of the data that support the findings of this study are available in the main text or Supplementary Information.

## Supplementary materials

Supplementary material 1 List of specimens examined Data type xls Explanation note List of all 2209 examined specimens of the megachilid bees from the Nakhchivan Autonomous Republic of Azerbaijan. https://binary.pensoft.net/file/1485327This dataset is made available under the Open Database License (http://opendatacommons.org/licenses/odbl/1.0/). The Open Database License (ODbL) is a license agreement intended to allow users to freely share, modify, and use this Dataset while maintaining this same freedom for others, provided that the original source and author(s) are credited.Alexander V. Fateryga, Mahir M. Maharramov, Maxim Yu. Proshchalykin
